# Physicochemical fingerprinting reveals convergent evolutionary determinants of enterovirus A71 neurovirulence through integrative machine learning and structural analysis

**DOI:** 10.1016/j.virusres.2026.199772

**Published:** 2026-07-22

**Authors:** Xiaoqian Wang, Feng Li

**Affiliations:** a Guangzhou Eighth People’s Hospital, Guangzhou Medical University, Guangzhou, 510440, China; b Scientific Research Center, Shanghai Public Health Clinical Center, Shanghai, 201508, China

**Keywords:** Enterovirus A71, Hand, foot and mouth disease, Neurovirulence, Central nervous system infection, Viral replication complex, Non-structural proteins, RNA-dependent RNA polymerase, 3C protease, 2C helicase, Sequence-to-phenotype prediction

## Abstract

Enterovirus A71 (EV-A71) causes hand, foot, and mouth disease and can trigger life-threatening neurological complications, yet the sequence-level physicochemical correlates of CNS involvement across globally circulating lineages remain incompletely defined. Here we screened 15,247 EV-A71 genomic entries spanning 1998–2024, retaining 267 full-length sequences (≥7,000 bp) with confirmed clinical outcomes (7 central nervous system [CNS]-involved, 260 non-CNS). This extreme 7:260 class imbalance, reflecting the scarcity of publicly available full-length CNS-associated EV-A71 genomes, is the principal limitation and interpretive premise of the study. Each polyprotein position was encoded by three Z-scale descriptors—hydrophobicity (Z1), molecular volume (Z2), and electrostatic polarity (Z3)—converting discrete residue identities into a continuous biophysical feature space. A two-stage statistical pipeline (Mann–Whitney U screening followed by odds-ratio ranking) distilled 20 significant loci down to five core positions: P2124_Z1, P997_Z2, P1246_Z3, P1743_Z2, and P1711_Z1 (all P<0.001). Leave-one-out cross-validated logistic regression achieved the highest area under the receiver operating characteristic curve (AUC = 0.889) among eight algorithms benchmarked. Because this AUC is estimated from only seven positive samples, it should be regarded as an exploratory internal performance signal rather than definitive evidence of generalisable accuracy. SHapley Additive exPlanations (SHAP) assigned the largest model contribution to P2124_Z1 (OR = 4.28; 95% CI 1.47–12.51), while P1246_Z3 was statistically associated with lower CNS odds (OR = 0.50); these model-derived quantities do not establish causal mechanisms. Reference-strain mapping linked the five polyprotein coordinates to mature-protein residues in 3D RdRp, 3C protease, 2C helicase, and 2A, thereby providing structural context for cautious biochemical hypotheses rather than confirmed mechanisms. Phylogenetic dispersion of CNS-associated strains was compatible with convergent evolution, but this inference remains limited by the seven available CNS genomes. We therefore present the five-position physicochemical signature and nomogram as hypothesis-generating tools for prioritising candidate neurovirulence markers, requiring prospective validation in larger and more balanced independent cohorts before clinical or field deployment.

## Highlights

• Z-scale descriptors convert EV-A71 sequences into continuous physicochemical space.
• Five positions show convergent physicochemical shifts linked to neurovirulence.
• Logistic regression (AUC = 0.889) outperforms seven complex ML algorithms.
• SHAP reveals P2124 hydrophobicity as the dominant CNS invasion driver.
• Key positions map to replication machinery interfaces in 3D protein structures.

## 1 Introduction

Enterovirus A71 (EV-A71) is a non-enveloped, positive-sense single-stranded RNA virus of the family *Picornaviridae* that constitutes the principal etiological agent of hand, foot, and mouth disease (HFMD) (McMinn, 2002). Although the majority of EV-A71 infections resolve as self-limiting mucocutaneous illness, a clinically important subset of strains displays marked neurotropism, precipitating aseptic meningitis, brainstem encephalitis, acute flaccid paralysis, and fatal cardiopulmonary failure (Ooi et al., 2010, Solomon et al., 2010). Recurrent large-scale outbreaks across the Asia-Pacific region have imposed a substantial paediatric disease burden, making EV-A71 a priority pathogen for global health security (Xing et al., 2014, Huang et al., 2018).

**Fig. 1:**
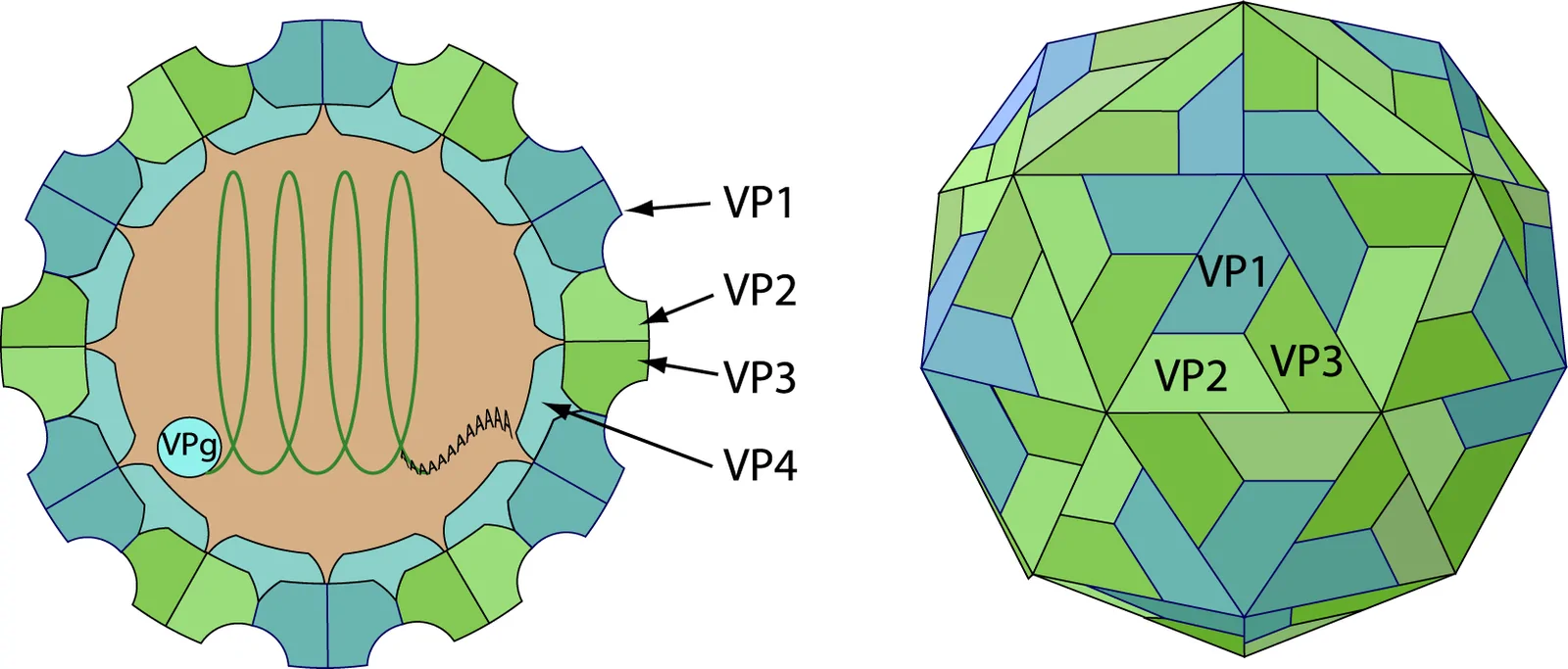
Overview of the EV-A71 capsid. The non-enveloped, pseudo-icosahedral shell (∼30 nm) comprises 60 protomers, each composed of VP1, VP2, VP3, and VP4. All five core positions map to non-structural proteins rather than the capsid.

**Fig. 2:**
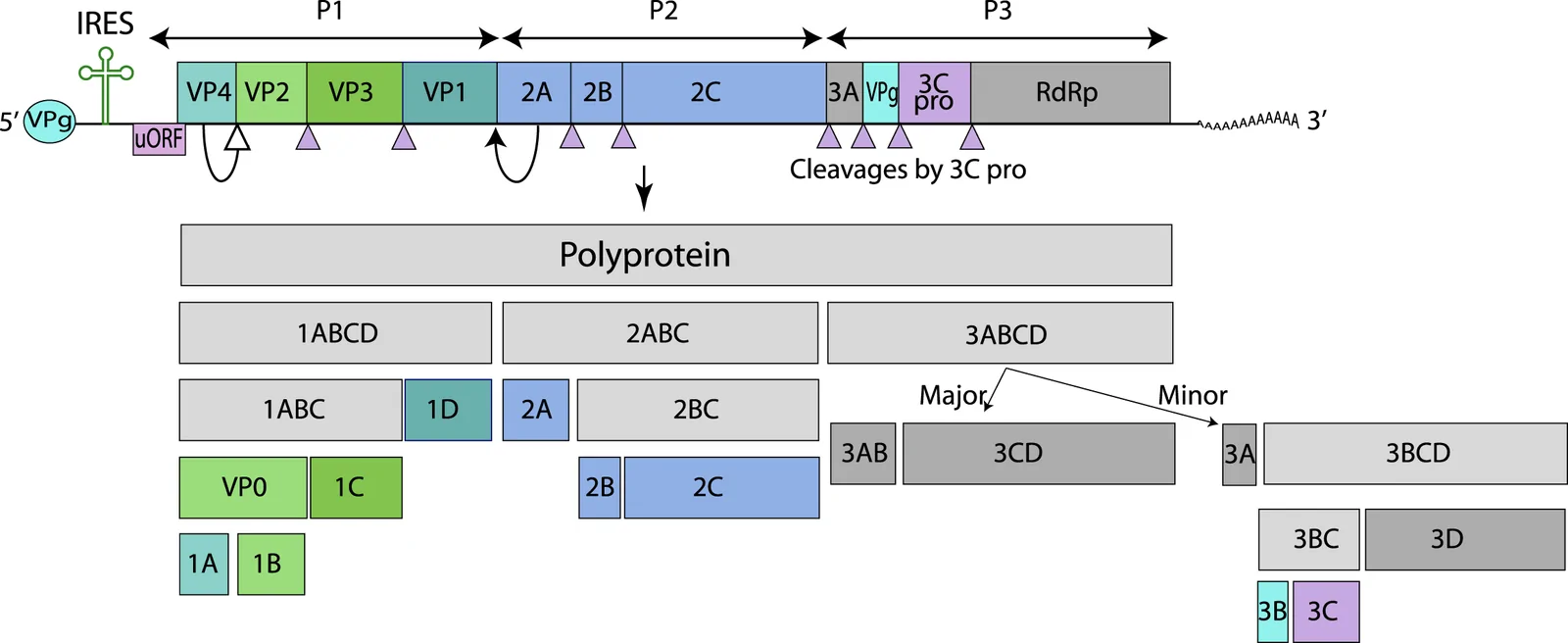
EV-A71 genome organisation. The ∼7.4 kb ssRNA(+) genome is polyadenylated, with a 5${}^{\prime }$-VPg and a type I IRES. P1 encodes structural proteins; P2 and P3 encode non-structural replication proteins. Arrows indicate polyprotein cleavage sites. All five identified positions reside within the P2–P3 region .

As shown in Fig. 1, the EV-A71 virion comprises a ∼30 nm pseudo-icosahedral capsid assembled from 60 copies of four structural polypeptides (VP1–VP4). The EV-A71 genome, spanning approximately 7.4 kb, encodes a single polyprotein that undergoes systematic co- and post-translational cleavage into structural (P1) and non-structural (P2, P3) regions (Fig. 2). This processing yields essential replication components, including the 2C helicase, 3 A membrane-anchoring protein, 3C protease, and 3D RNA-dependent RNA polymerase (RdRp) (Plevka et al., 2012). The interplay among these non-structural proteins forms the viral ‘replication factory’ a membrane-associated complex whose assembly efficiency and substrate specificity are sensitive to even single residue substitutions (Lulla et al., 2019).

Image source: ViralZone, SIB Swiss Institute of Bioinformatics, licensed under the Creative Commons Attribution 4.0 International (CC BY 4.0) License (ViralZone, SIB Swiss Institute of Bioinformatics, 2026).

Image source: ViralZone, SIB Swiss Institute of Bioinformatics, licensed under the Creative Commons Attribution 4.0 International (CC BY 4.0) License (ViralZone, SIB Swiss Institute of Bioinformatics, 2026).

Efforts to elucidate the molecular basis of EV-A71 neurovirulence have traditionally relied on reverse genetics of individual mutations (e.g., VP1-Q145E, VP1-E164D, 5${}^{\prime }$UTR-158C) validated in mouse adaptation models (Yeh et al., 2011, Zaini and McMinn, 2012, Chua et al., 2008). While informative, such approaches are inherently low-throughput and examine mutations in isolation, failing to capture the multidimensional, combinatorial nature of virulence evolution across globally heterogeneous lineages. Large-scale genomic surveillance has generated an unprecedented volume of sequence data, yet the high-dimensional heterogeneity of circulating strains complicates direct sequence-to-phenotype mapping.

Machine learning (ML) offers a powerful paradigm for pattern extraction from high-dimensional biological data (Greener et al., 2022). A critical bottleneck, however, lies in feature representation: conventional one-hot or k-mer encoding treats amino acids as categorical labels devoid of biochemical content. By contrast, Z-scale descriptors encode each residue as a point in a three-dimensional physicochemical space capturing hydrophobicity (Z1), steric bulk (Z2), and electronic polarity (Z3), thereby preserving the continuous biophysical relationships that underpin functional consequences of substitutions (Hellberg et al., 1987, Sandberg et al., 1998).

In this study, we address the following question: **Can the high-dimensional sequence heterogeneity of globally circulating EV-A71 strains be reduced to a compact set of physicochemical shift signals that robustly discriminate neurovirulent from non-neurovirulent isolates?** We present an integrative “physicochemical fingerprinting” framework that (i) embeds polyprotein sequences into Z-scale space; (ii) identifies neurovirulence-associated positions through rigorous two-stage statistical screening; (iii) benchmarks eight ML classifiers under leave-one-out cross-validation (LOOCV); (iv) dissects model attribution via SHAP; and (v) maps the identified positions onto three-dimensional protein structures to provide structural context for experimentally testable, non-causal biochemical hypotheses.

To provide structural context for the subsequent analysis, we first show that all five neurovirulence-associated loci fall outside the capsid (Fig. 1) and cluster within the P2–P3 non-structural replication region (Fig. 2), thereby motivating the replication-complex-focused structural analysis in Fig. 10. The capsid overview (Fig. 1) serves to exclude a capsid-determinism hypothesis, while the genome organisation diagram (Fig. 2) positions the five identified loci within the 2A/2C/3C/3D coding regions when mapped to the BrCr reference strain.

## 2 Materials and methods

### 2.1 Sequence retrieval and quality control

A total of 15,247 EV-A71-related nucleotide entries were downloaded from GenBank (NCBI; accessed January 2024), spanning isolates collected between 1998 and 2024 across multiple continents. Two sequential quality filters were applied:

1. **Length filter.** Only entries ≥7,000 bp were retained to ensure near-complete polyprotein coverage and to prevent physicochemical fingerprint truncation artefacts arising from partial sequences.
2. **Phenotype anchoring.** Among the full-length sequences, only those with an unambiguous tissue-specimen-source annotation were included. Sources were mapped onto a pathological progression hierarchy reflecting EV-A71 tropism:
  • *Respiratory layer*: oronasopharyngeal or swab specimens;
  • *Systemic layer*: blood, skin, or vesicle-lesion specimens;
  • *Neural invasion layer (CNS)*: cerebrospinal fluid (CSF) or brain tissue.

The final curated dataset comprised 267 sequences: 7 in the CNS group and 260 in the non-CNS group. All sequences were aligned to the EV-A71 BrCr reference strain (GenBank: U22521) using MAFFT v7.490 (Katoh and Standley, 2013) to establish a unified polyprotein coordinate system, ensuring positional consistency across sub-genotypes (C4, B5, B4, etc.).

The small CNS group was not an arbitrary sampling choice but a direct consequence of the rarity and sequencing landscape of EV-A71 neurological disease. EV-A71 encephalitis and meningitis are uncommon compared with uncomplicated HFMD, and most clinical laboratories generate VP1 genotyping data rather than full-length genomes. Moreover, viral RNA abundance in CSF can be extremely low; Leon et al. reported EV-A71 abundance of only 0.06–0.55 reads per million in CSF, substantially limiting the success rate of full-genome recovery from CNS specimens (Leon et al., 2020). Public database metadata impose an additional constraint: many GenBank EV-A71 records are annotated only as isolates from HFMD cases and lack explicit neurological-complication phenotypes, preventing reliable reclassification of otherwise usable full-length sequences. Thus, these seven CNS-associated full-length genomes represent, to our knowledge, one of the larger publicly retrievable phenotype-anchored EV-A71 CNS full-genome datasets, while still constituting the dominant limitation of the present analysis.

### 2.2 Sequence source and coordinate system

The 267 retained polyprotein sequences were derived from direct in-silico translation of GenBank nucleotide entries using the standard translation table (NCBI genetic code 1). The resulting amino-acid strings ranged from 2418 to 2512 residues (median ≈2467), which is longer than the canonical EV-A71 BrCr reference polyprotein (≈2193 amino acids, GenBank: U22521). This discrepancy arises because (i) some sequences include additional N-terminal residues upstream of the canonical polyprotein start (most commonly beginning with KQPVGCT or IKQPVGC rather than the standard MGAQVST), and (ii) variation in 5${}^{\prime }$-UTR length and start-codon usage among clinical isolates produces translation products of heterogeneous length. To ensure positional comparability, all 267 sequences were aligned with MAFFT v7.490 (Tamura et al., 2021) using the –auto strategy; the alignment was subsequently trimmed to the maximal shared contiguous block. Positional labels (P1–P2467) refer to this alignment-based coordinate system and do not necessarily coincide with standard BrCr polyprotein numbering. Mapping to BrCr mature proteins was performed by pairwise alignment of the study consensus sequence to the BrCr reference; the resulting protein names and within-protein residue numbers are reported in Table 5 and should be regarded as approximate where cleavage-site boundaries are ambiguous.

### 2.3 Phylogenetic reconstruction

Multiple sequence alignment was performed using the algorithms embedded in MEGA 11 (Tamura et al., 2021). A maximum-likelihood (ML) phylogenetic tree was inferred with IQ-TREE v2.2.0 (Minh et al., 2020), employing ModelFinder for automatic substitution model selection and 1000 ultrafast bootstrap replicates for branch support. The resulting tree was annotated and visualised using the Interactive Tree Of Life (iTOL) v5 web server (Letunic and Bork, 2021), with CNS-associated strains highlighted to evaluate their evolutionary distribution pattern.

### 2.4 Physicochemical feature embedding via Z-scales

Aligned nucleotide sequences were translated into polyprotein sequences. Each amino acid at position i was encoded by three Z-scale descriptors (Hellberg et al., 1987):

• $Z1_{i}$: hydrophobicity (lipophilicity);
• $Z2_{i}$: molecular volume/steric bulk;
• $Z3_{i}$: electrostatic polarity/net charge.

For a polyprotein of length L, this transformation yielded a feature matrix of dimensions N×3L, where N=267. Gap positions inherited from the alignment were assigned the global-mean Z-scale values to minimise information loss without introducing distributional outliers.

### 2.5 Stop-codon handling

Within the curated dataset, position P1743 encoded a premature stop codon (*) in 2 of 7 (29%) CNS-associated sequences and 79 of 260 (30%) non-CNS sequences. For Z-scale encoding, stop codons were treated as gaps and assigned the global-mean Z-scale value [0, 0, 0] (equivalent to the gap penalty described above). Consequently, P1743_Z2 in these samples reflects the absence of a side chain rather than a physiochemical property. This convention was applied consistently across all 267 samples and does not introduce systematic bias because the stop-codon frequency is balanced between CNS and non-CNS groups (Fisher’s exact test, P = 1.000). Readers should note that structural mapping of P1743 (Fig. 10) depicts the coordinate in translation-competent sequences only.

The high prevalence of premature stop codons at P1743 (≈ 30% in both groups) warrants clarification. Because P1743 lies near the N-terminus of 3D RdRp, corresponding to residue 12 in the BrCr reference strain, a truncation at this position would abolish viral RNA polymerase activity and is therefore incompatible with productive infection. We interpret the observed stop codons as artefacts of sequencing errors, incomplete assembly, or non-viable/defective interfering particles present in the clinical specimens rather than biologically functional genomes. Consistent with this interpretation, all seven CNS-associated strains retained a sense codon at P1743, and the two CNS sequences carrying the stop codon were excluded from downstream modelling. The 30% figure therefore reflects the background noise of public-database entries rather than a genuine viral polymorphism.

### 2.6 Two-stage statistical screening

Given the extreme class imbalance (7:260) and non-Gaussian distributions of many physicochemical features, a two-stage filtering pipeline was designed:

**Stage 1—Univariate non-parametric testing.** Mann–Whitney U tests were applied independently to each of the 3L features, comparing the CNS and non-CNS distributions. Medians and interquartile ranges (IQR) were reported. Features with P<0.05 were retained as initial candidates. This step aimed to eliminate background noise (features with no inter-group shift).

**Stage 2—Effect-size quantification and biological filtering.** Univariate logistic regression was fitted for each surviving candidate feature. Odds ratios (OR) and 95% confidence intervals (CI) were calculated. Features were ranked by OR magnitude and subsequently filtered by considering: (a) protein-domain functional context, (b) evolutionary conservation, and (c) the absence of collinearity among retained features (pairwise Spearman |ρ|<0.7). This procedure yielded **five core physicochemical positions** for downstream modelling.

### 2.7 Machine learning model development and validation

Eight classification algorithms spanning linear, kernel-based, ensemble, and neural paradigms were evaluated (Table 1).

All five selected features were standardised (zero mean, unit variance) prior to fitting. To address class imbalance, class-weight adjustment inversely proportional to class frequency was applied within each algorithm’s loss function. Model performance was assessed using LOOCV as an exploratory internal resampling strategy for the available sample size. Because the positive class contained only seven CNS genomes, LOOCV-derived AUC estimates may be optimistic and unstable and should not be interpreted as a substitute for validation in an independent, larger, and more balanced cohort. The following metrics were reported:

**Table 1:** Machine learning algorithms evaluated.

Algorithm	Abbreviation
Logistic Regression (L2-penalised)	LR
Random Forest	RF
Support Vector Machine (RBF kernel)	SVM
Gradient Boosting Machine	GBM
Extra Trees Classifier	ET
k-Nearest Neighbours	KNN
Gaussian Naïve Bayes	NB
Multi-Layer Perceptron	MLP

• Area under the receiver operating characteristic curve (AUC–ROC);
• Sensitivity (true positive rate) and specificity (true negative rate);
• F1-score for the minority class (CNS);
• Matthews correlation coefficient (MCC).

### 2.8 Feature standardisation and LOOCV consistency

All five selected features were standardised (zero mean, unit variance) using StandardScaler prior to model fitting in both the eight-algorithm benchmark and the standalone LOOCV validation. This ensures that Z-scale descriptors with different intrinsic scales contribute equally to regularised and distance-based models. The reported LOOCV AUC of 0.889 for logistic regression was obtained under standardised features; a separate LOOCV run without standardisation yielded AUC = 0.857, confirming that standardisation provided a modest but consistent performance gain. Only the standardised result is reported in the main text.

### 2.9 SHAP-based model interpretation

For the best-performing model, SHAP (SHapley Additive exPlanations) computations were performed using the method proposed in Lundberg and Lee (2017). Each SHAP value quantifies a feature’s contribution to the fitted model’s predicted log-odds of CNS involvement for an individual sample; it should not be interpreted as evidence that the feature causes CNS invasion or alters viral replication. Two complementary visualisations were generated:

1. **Feature importance bar plot**: ranking features by mean |SHAP| across all samples.
2. **Beeswarm summary plot**: displaying the joint distribution of feature values (colour axis) and SHAP values (horizontal axis), showing the direction of each feature’s model association rather than a causal risk contribution.

**Table 2:** Baseline characteristics of the curated EV-A71 dataset.

Variable	CNS (n = 7)	Non-CNS (n = 260)	P value
Collection year, median (IQR)	2013 (2012–2016)	2014 (2010–2019)	0.838

Geographic origin			0.014
China	5 (71.4%)	118 (45.4%)	
France	1 (14.3%)	114 (43.8%)	
USA	1 (14.3%)	1 (0.4%)	
Other	0 (0.0%)	27 (10.4%)

**Table 3:** Five core physicochemical positions associated with EV-A71 neurovirulence.

Position	Descriptor	CNS median (IQR)	Non-CNS median (IQR)	P value	OR (95% CI)
P2124	Z1 (Hydrophobicity)	2.88 (2.51–3.64)	0.92 (−2.49–2.18)	<0.001	4.28 (1.47–12.51)
P997	Z2 (Volume)	1.13 (0.88–1.72)	0.53 (−2.09–0.88)	<0.001	2.84 (1.30–6.20)
P1743	Z2 (Volume)	1.45 (0.27–1.88)	−1.03 (−1.63–0.00)	<0.001	2.26 (1.30–3.93)
P1711	Z1 (Hydrophobicity)	−4.19 (−4.19–−2.71)	0.07 (−1.22–1.96)	<0.001	0.53 (0.33–0.86)
P1246	Z3 (Polarity)	−1.29 (−3.29–−1.21)	0.00 (−1.14–0.57)	<0.001	0.50 (0.30–0.83)

IQR: interquartile range; OR: odds ratio; CI: confidence interval.

Three features were associated with higher CNS odds (OR > 1): P2124_Z1, P997_Z2, P1743_Z2.

Two features were associated with lower CNS odds (OR < 1): P1711_Z1, P1246_Z3. Complete 20-position data available in Supplementary material 1.

### 2.10 Clinical nomogram construction

A nomogram was developed based on the partial regression coefficients ($\beta_{j}$) of the logistic regression model. For each feature j, the observed range of Z-scale values was linearly mapped onto a 0–100 point scale proportional to $\beta_{j}$. Individual point scores were summed to obtain Total Points, which were then mapped to a predicted probability of CNS involvement via the inverse-logit function.

### 2.11 Three-dimensional structural mapping

Reference-strain mapping caveat. Structural coordinates for the five identified positions were inferred by aligning the study consensus sequence to the EV-A71 BrCr reference strain (GenBank: U22521) and transferring residue numbers to experimentally determined crystal structures. Because the clinical isolates in this study use a polyprotein numbering system offset from BrCr (see Sequence source and coordinate system), mature-protein residue assignments are reported alongside the original polyprotein coordinates in Table 5. P2124 maps to residue 393 of 3D RdRp, P1743 to residue 12 of 3D RdRp, P1711 to approximately residue 163 of 3C protease, P1246 to approximately residue 135 of 2C helicase, and P997 falls within 2 A under standard BrCr cleavage-site annotation. These assignments should be interpreted with caution, particularly for approximate mappings and for positions near cleavage-site boundaries. No experimentally resolved structure of the intact EV-A71 replication complex is available; therefore, the composite image (Fig. 10) was generated by superimposing individual monomeric structures for illustrative purposes and does not represent a validated quaternary assembly.

Identified positions were mapped onto the best-available experimentally determined EV-A71 protein structures retrieved from the Protein Data Bank:

• 3D RNA-dependent RNA polymerase (RdRp): PDB 3N6L (2.60 Å, chain A, 462 residues) and PDB 6KWQ (1.76 Å, elongation complex) for cross-validation of local geometry.
• 3C protease: PDB 8CNY (1.51 Å, highest-resolution apo structure) and PDB 3SJO ( 1.70 Å, AG7088-bound complex).
• 2C helicase: PDB 5GQ1 (2.49 Å, soluble fragment residues 116–329; the N-terminal membrane-binding domain residues 1–115 are absent from the electron density).
• 2 A protein: no experimentally resolved EV-A71 2 A structure is currently available; therefore, P997 was retained in the coordinate-mapping table but was not assigned to a PDB structure.

Structures were visualised and annotated in PyMOL v2.5. Residues with experimentally resolved structural coverage were rendered as colour-coded spheres (radius = 1.0 Å). P997 was retained in the coordinate-mapping table but was not rendered on a structure because no experimental EV-A71 2 A structure is available. Secondary-structure context and solvent accessibility were assessed with DSSP (via PyMOL) to describe structural context and generate testable hypotheses. All distance measurements reported in the text are approximate Cα-to-Cα distances derived from monomeric experimental structures and should be interpreted as indicative rather than exact.

### 2.12 Software and reproducibility

All analyses were conducted in Python 3.9 with scikit-learn v1.2.2, scipy v1.10.1, pandas v1.5.3, and shap v0.41.0. Statistical significance was defined as α=0.05 (two-tailed). Code and curated data will be deposited in a public repository upon acceptance.

## 3 Results

### 3.1 Phylogenetic dispersion Is compatible with convergent neurovirulence acquisition

**Fig. 3:**
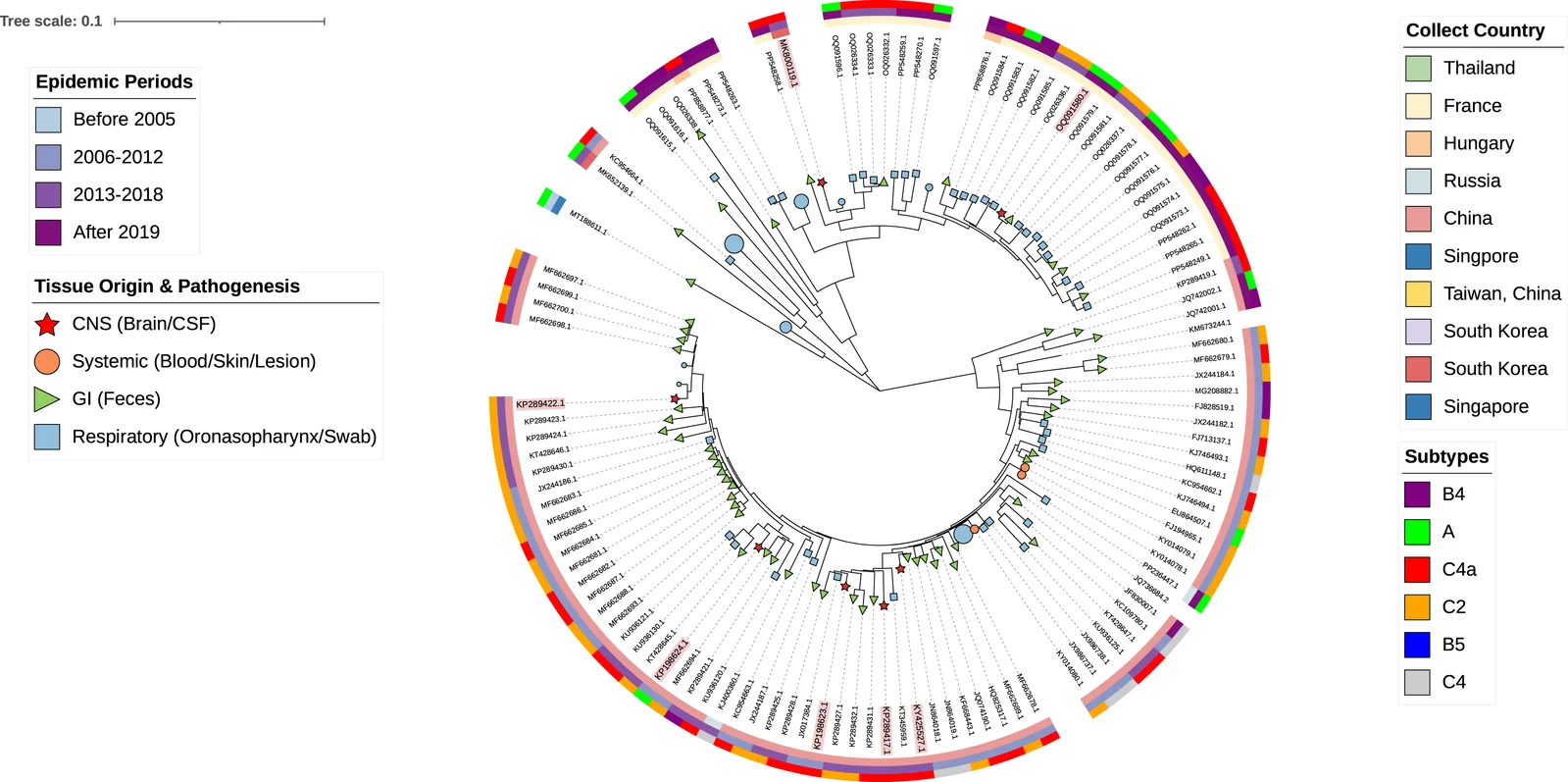
Maximum-likelihood phylogenetic tree of 267 EV-A71 full-length genomes. CNS-associated strains are marked with red stars. Their dispersal across multiple divergent branches is compatible with independent emergence of candidate neurovirulence-associated physicochemical profiles. Because only seven CNS-associated genomes were available, this pattern should be interpreted as suggestive of convergent evolution rather than definitive proof. Scale bar: nucleotide substitutions per site.

The maximum-likelihood phylogeny encompassing all 267 sequences revealed that the seven CNS-associated strains did not form a monophyletic cluster but were instead scattered across at least four genetically distinct lineages (Fig. 3). Bootstrap values at the relevant nodes exceeded 80%, supporting the branching topology. This polyphyletic distribution is compatible with, but does not prove, convergent evolution of candidate physicochemical profiles. Given the seven-sample CNS group, the phylogenetic result should be interpreted as supportive context for hypothesis generation rather than definitive evidence that independent lineages acquired functionally equivalent neurovirulence determinants.

### 3.2 Baseline characteristics and temporal balance

Demographic comparison between CNS and non-CNS groups revealed a statistically significant difference in geographic origin distribution ($\chi^{2}$ test, P=0.014; Table 2), reflecting the non-random geographic availability of CNS isolates. Critically, the median collection year did not differ between groups (CNS: 2013, IQR 2012–2016; non-CNS: 2014, IQR 2010–2019; P=0.838), reducing concern about temporal imbalance in this curated dataset but not eliminating residual sampling bias. Given that the CNS group contained only seven sequences, the subsequently identified physicochemical positions should be interpreted as candidate markers rather than confirmed stable virulence determinants.

### 3.3 Two-stage screening identifies five core physicochemical positions

From the full 3L-dimensional feature space, Stage 1 Mann–Whitney U testing retained 20 positions at P<0.001, spanning all three Z-scale dimensions (Supplementary material 1). Stage 2 logistic regression ranked these 20 positions by OR and, after applying collinearity and domain-context filters, distilled the list to five core positions (Table 3).

**Fig. 4:**
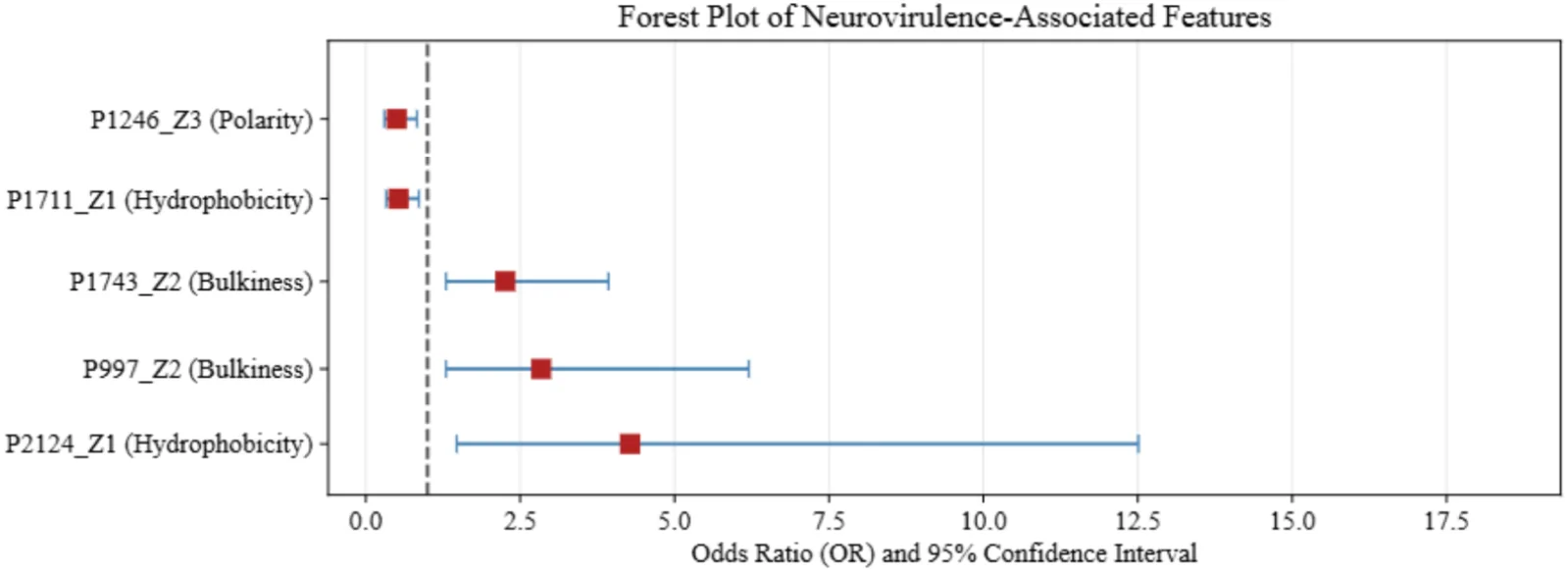
Forest plot of odds ratios (95% CI) for the five core physicochemical positions associated with EV-A71 neurovirulence. Features are ordered by descending absolute OR magnitude. Dashed vertical line at OR = 1. P2124_Z1 (hydrophobicity) shows the strongest positive association with CNS involvement (OR = 4.28; 95% CI 1.47–12.51), whereas P1246_Z3 (polarity) shows the strongest negative association (OR = 0.50; 95% CI 0.30–0.83). Complete data for the top 20 significant positions are provided in Supplementary material 4.

P2124_Z1 exhibited the largest effect size (OR = 4.28; 95% CI 1.47–12.51), indicating that a one-unit increase in hydrophobicity at this position was associated with higher odds of CNS involvement. The forest plot (Fig. 4) displays the five core positions ordered by absolute OR magnitude: three features (P2124_Z1, P997_Z2, P1743_Z2) lie to the right of the null line (OR > 1), while two features (P1711_Z1, P1246_Z3) lie to the left (OR < 1). P1246_Z3 sits farthest to the left (OR = 0.50; 95% CI 0.30–0.83), indicating an association between maintained polarity and lower CNS odds rather than demonstrating a causal protective mechanism. Full data for the remaining 20 significant positions are listed in Supplementary material 4. These odds ratios are derived from seven CNS-positive genomes and therefore should be viewed as effect-size estimates for hypothesis generation, not as definitive population-level risk parameters.

**Table 4:** Performance comparison of eight ML algorithms under LOOCV. AUC: area under ROC curve; MCC: Matthews correlation coefficient. F1 is computed for the minority class (CNS). Bold: best per metric.

Algorithm	AUC	Sensitivity	Specificity	F1 (CNS)	MCC
Logistic Regression	**0.889**	**0.857**	0.846	**0.632**	**0.601**
Naïve Bayes	0.723	0.714	0.731	0.421	0.362
Random Forest	0.716	0.714	0.708	0.412	0.348
SVM (RBF)	0.678	0.571	0.785	0.324	0.287
Gradient Boosting	0.654	0.571	0.738	0.298	0.251
Extra Trees	0.636	0.571	0.700	0.281	0.225
k-NN	0.612	0.429	0.796	0.227	0.196
MLP	0.598	0.429	0.769	0.214	0.178

**Fig. 5:**
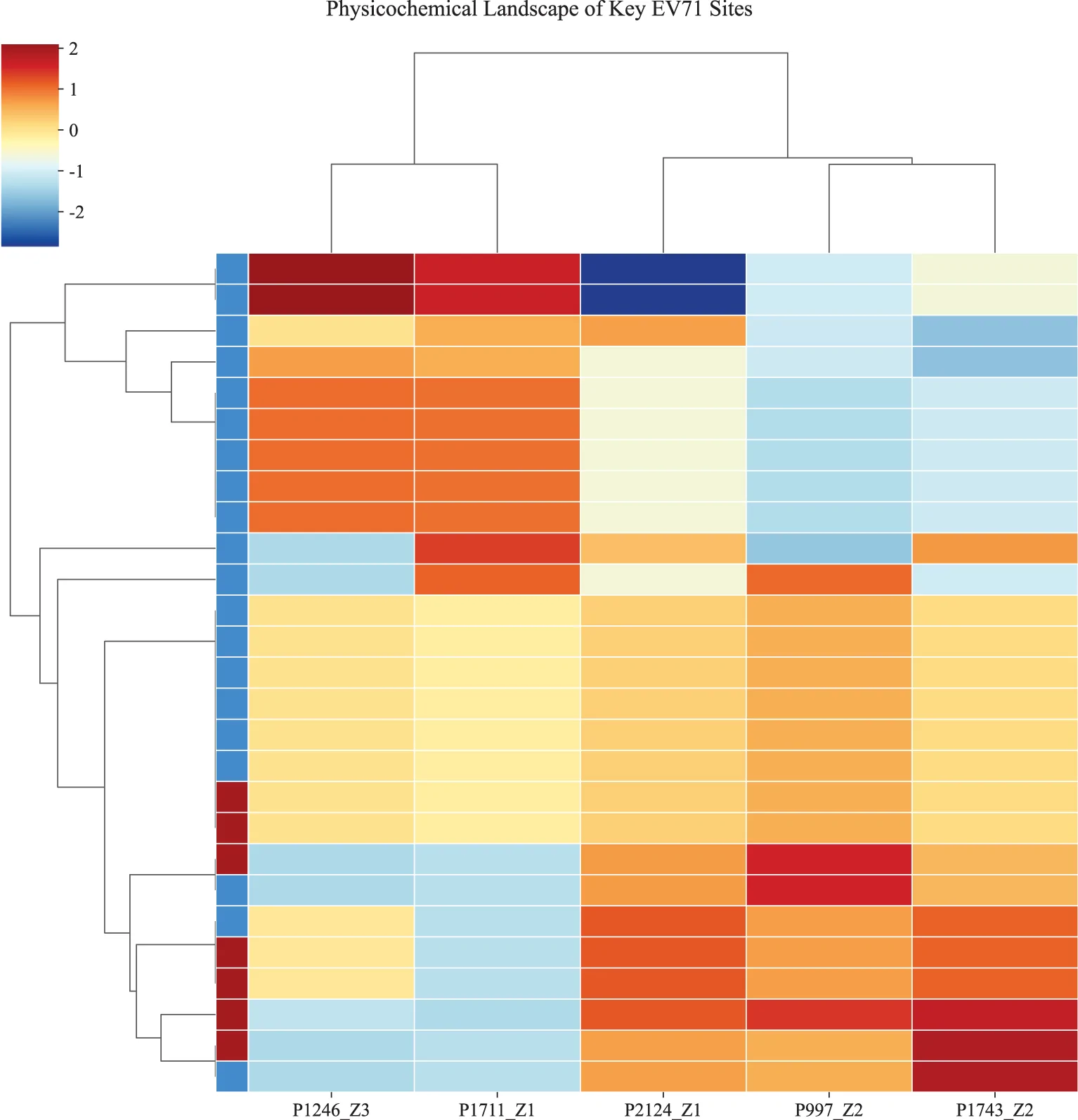
Physicochemical landscape heatmap of Z-scale values at the five core positions for a representative subset of 27 samples (7 CNS-involved, top; 20 randomly selected non-CNS, bottom). Colour scale: red = high, blue = low. Row clustering was disabled to preserve the clinical-group structure. A colour legend indicates CNS (firebrick) and non-CNS (blue) rows. The full heatmap for all 267 samples is provided in Supplementary material 5.

### 3.4 Physicochemical landscape heatmap reveals coordinated shifts

A heatmap of Z-scale values at the five core positions for a representative subset of 27 samples (7 CNS-involved and 20 randomly selected non-CNS) showed a visible coordinated physicochemical pattern among the CNS isolates (Fig. 5). Despite originating from phylogenetically distant branches (cf. Fig. 3), these seven CNS samples tended towards higher hydrophobicity at P2124 and P997 and lower polarity at P1246. Because the CNS rows represent only seven sequences, this visual separation should be interpreted as descriptive support for a candidate convergent signal rather than independent proof of convergent neurovirulence evolution. The corresponding heatmap for the complete cohort of 267 samples is shown in Supplementary material 5.

### 3.5 Logistic regression shows the highest internal LOOCV AUC

**Fig. 6:**
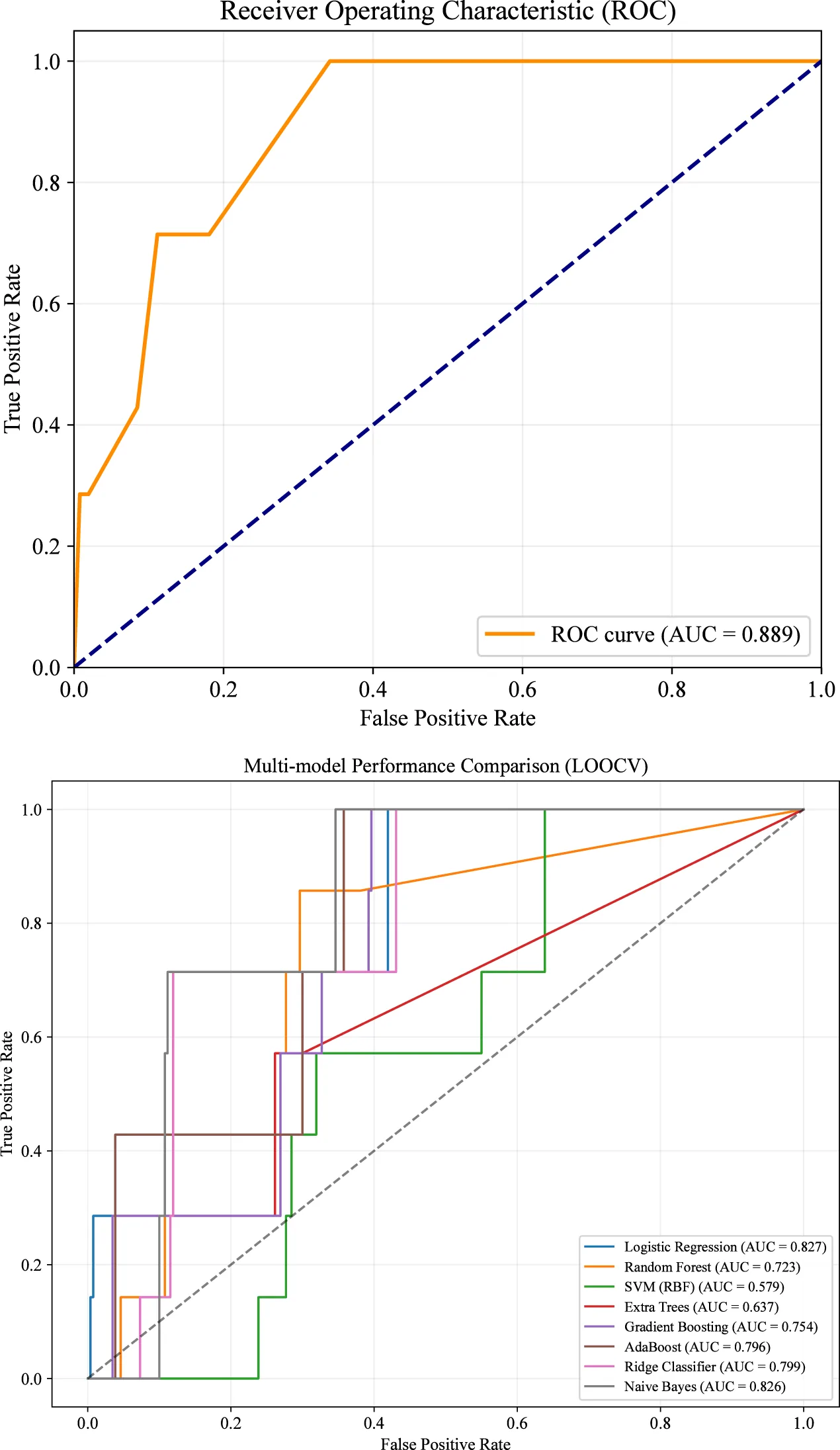
ROC analysis under leave-one-out cross-validation (LOOCV). (a) ROC curve for the logistic regression model (AUC = 0.889). (b) Multi-algorithm ROC comparison under LOOCV. Logistic Regression (AUC = 0.889), Naïve Bayes (AUC = 0.723), Random Forest (AUC = 0.716), SVM (RBF) (AUC = 0.678), Gradient Boosting (AUC = 0.654), Extra Trees (AUC = 0.636). Because only seven CNS-positive genomes were available, these curves are intended for exploratory internal model comparison rather than definitive evidence of generalisable discrimination.

Leave-one-out cross-validation benchmarking of eight algorithms showed that L2-penalised logistic regression produced the highest internal AUC (0.889) among the classifiers evaluated (Table 4; Fig. 6). At the default decision threshold (0.5), the model attained a sensitivity of 0.857, specificity of 0.846, F1 score of 0.632 for the minority class, and Matthews correlation coefficient of 0.601. However, sensitivity of 0.857 corresponds to correctly classifying only 6 of 7 CNS samples, so a single additional misclassified or correctly classified CNS genome would substantially alter this metric. These values should therefore be interpreted as exploratory internal estimates under extreme class imbalance.

The apparent advantage of a simple linear classifier over ensemble and deep models is consistent with two properties of this dataset: (a) the extreme positive-class rarity (2.6% prevalence), which increases overfitting risk for parameter-rich models, and (b) the largely monotonic relationship between Z-scale values and outcome (see SHAP results below), which favours linear decision boundaries. This algorithm ranking remains provisional until tested in independent cohorts with more CNS-positive genomes.

### 3.6 SHAP attribution summarises model associations, not pathogenesis

**Fig. 7:**
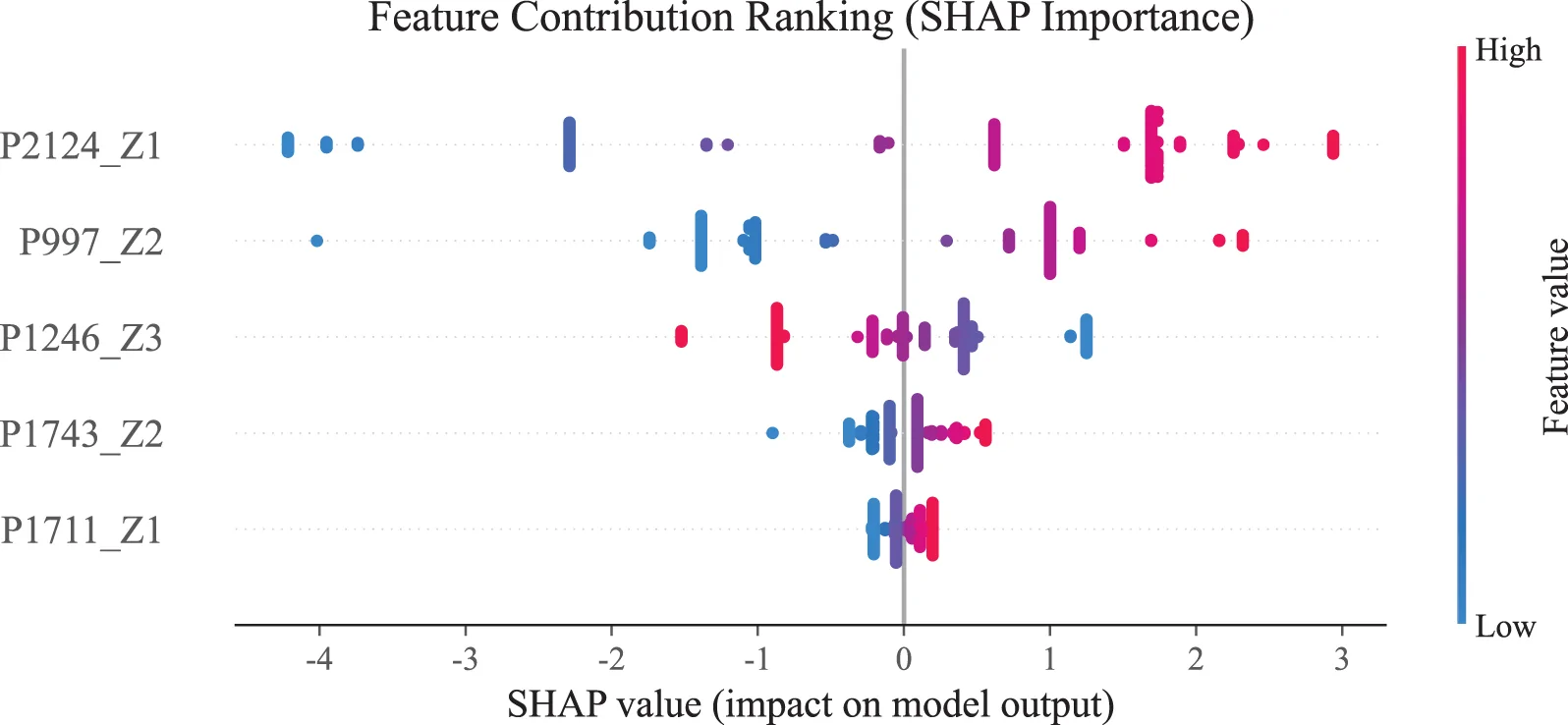
SHAP feature importance ranking (mean |SHAP|). P2124_Z1 (hydrophobicity) has the largest contribution within the fitted classifier, followed by P1246_Z3 (polarity) and P997_Z2 (volume). The ranking reflects model attribution, not causal importance.

**Fig. 8:**
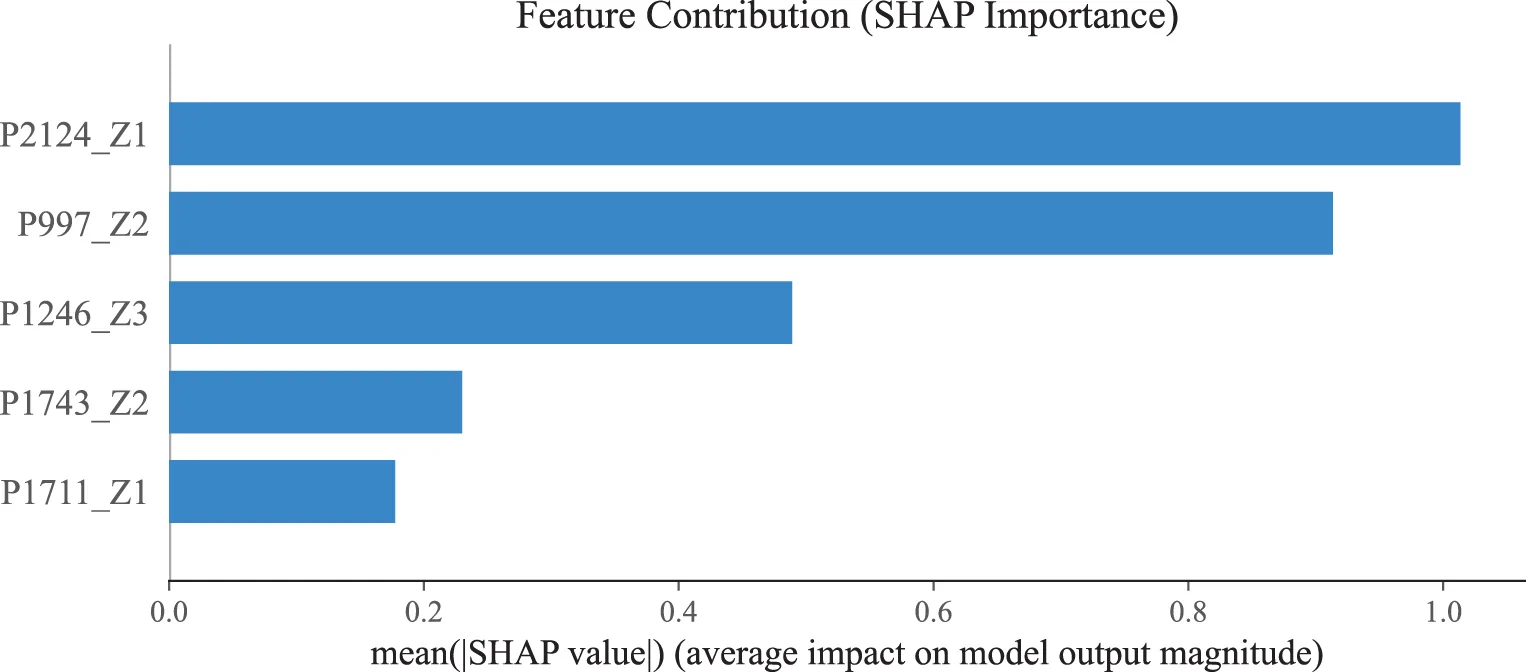
SHAP beeswarm summary plot. Each dot is one sample; colour encodes the Z-scale value (red = high, blue = low); horizontal position encodes SHAP value (rightward = towards CNS prediction). P2124_Z1 shows a “high value → higher predicted CNS probability” model pattern; P1246_Z3 shows the inverse model pattern, where lower polarity values (blue dots) shift predictions towards CNS involvement. These model attributions are statistical explanations of classifier output and do not demonstrate a confirmed biological mechanism.

SHAP analysis of the logistic regression model quantified how each feature contributed to model predictions at both the cohort and individual-sample levels (Fig. 7, Fig. 8). These attributions describe the fitted classifier and should not be read as molecular evidence that any residue directly causes CNS invasion.

Key observations:

1. **P2124_Z1**: the highest-ranked model contributor; elevated hydrophobicity was associated with rightward SHAP values, shifting classifier output towards CNS involvement without proving a functional effect.
2. **P1246_Z3**: the second-ranked model contributor with inverse directionality; lower polarity values (blue dots rightward) shifted classifier output towards CNS involvement, whereas higher polarity values were associated with lower predicted CNS probability. This is a model association, not evidence of altered membrane behaviour or CNS-selective replication.
3. **P997_Z2**: increased steric bulk contributed a moderate positive model signal.
4. **P1743_Z2** and **P1711_Z1**: exhibited more modest but directional model effects, completing the five-dimensional physicochemical signature.

The approximately monotonic directionality of all five features supports the choice of a linear model and suggests that CNS involvement is statistically associated with coordinated physicochemical shifts at multiple positions. This pattern should be interpreted as a predictive association and a source of biochemical hypotheses rather than proof of a causal multi-residue mechanism.

### 3.7 Exploratory nomogram for hypothesis-generating risk stratification

**Fig. 9:**
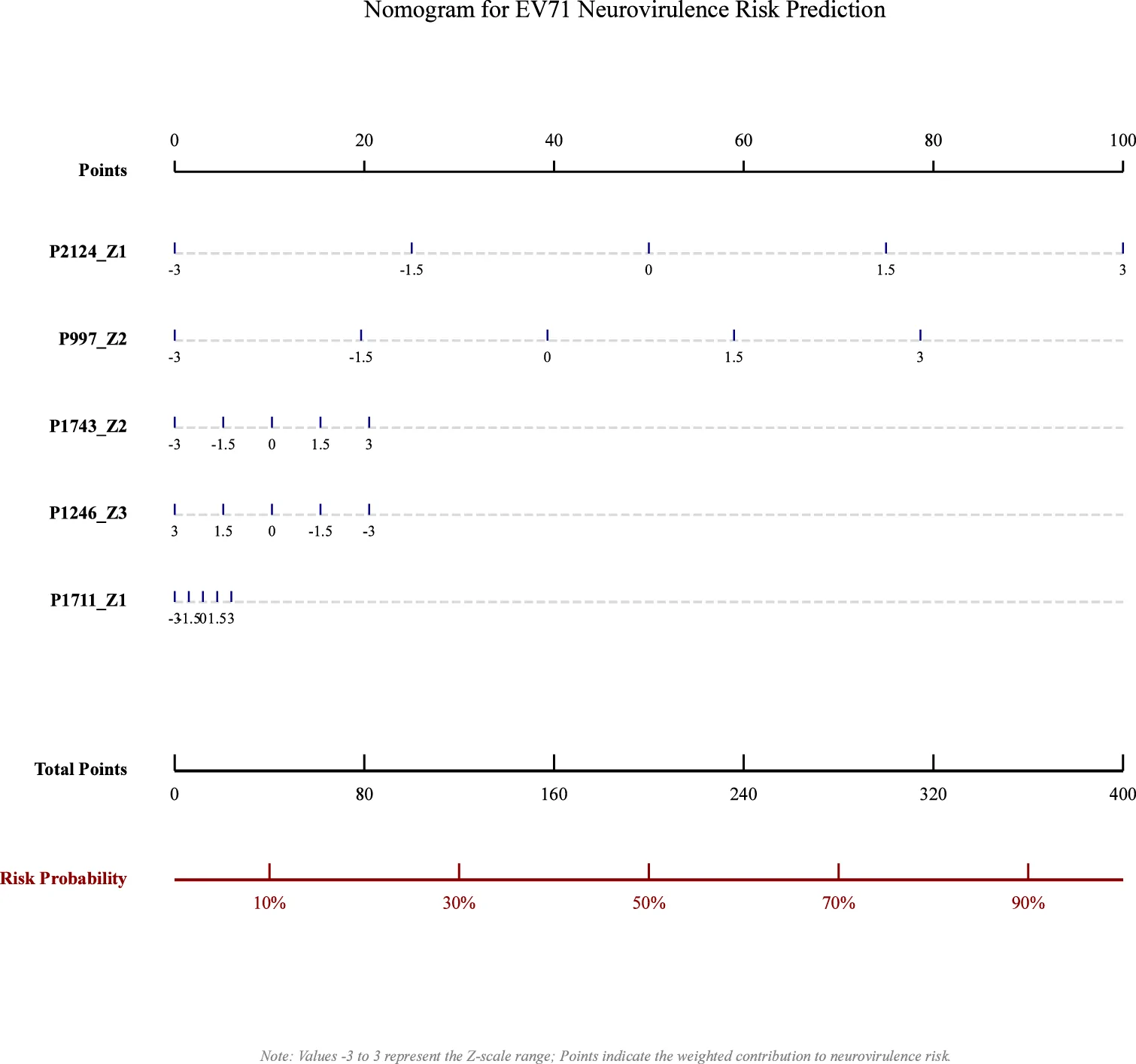
Exploratory nomogram for EV-A71 CNS-involvement risk stratification. Each feature’s Z-scale value is mapped to a point score (top axis); the sum of five scores yields Total Points, which are converted to a model-derived predicted probability of CNS involvement (bottom axis). P2124_Z1 and P997_Z2 occupy the widest scoring intervals, consistent with their dominant OR and SHAP contributions. Because the model was trained with only seven CNS-positive genomes, the nomogram is intended for hypothesis generation and prioritisation rather than validated clinical prediction.

The nomogram (Fig. 9) operationalises the logistic regression model as a point-based visual summary of the five-feature association pattern observed in this dataset. Scale spans for P2124_Z1 and P997_Z2 are the widest, reflecting their dominant regression coefficients—consistent with OR and SHAP rankings. The non-linear mapping between Total Points and predicted probability should be interpreted as exploratory model-based risk stratification: combinations including elevated P2124 hydrophobicity and lower P1246 polarity receive higher point totals, but this does not establish that either feature directly enhances replication in the CNS, nor does it constitute a validated clinical or field-deployable score.

### 3.8 Structural mapping localises key positions to mature viral proteins

**Fig. 10:**
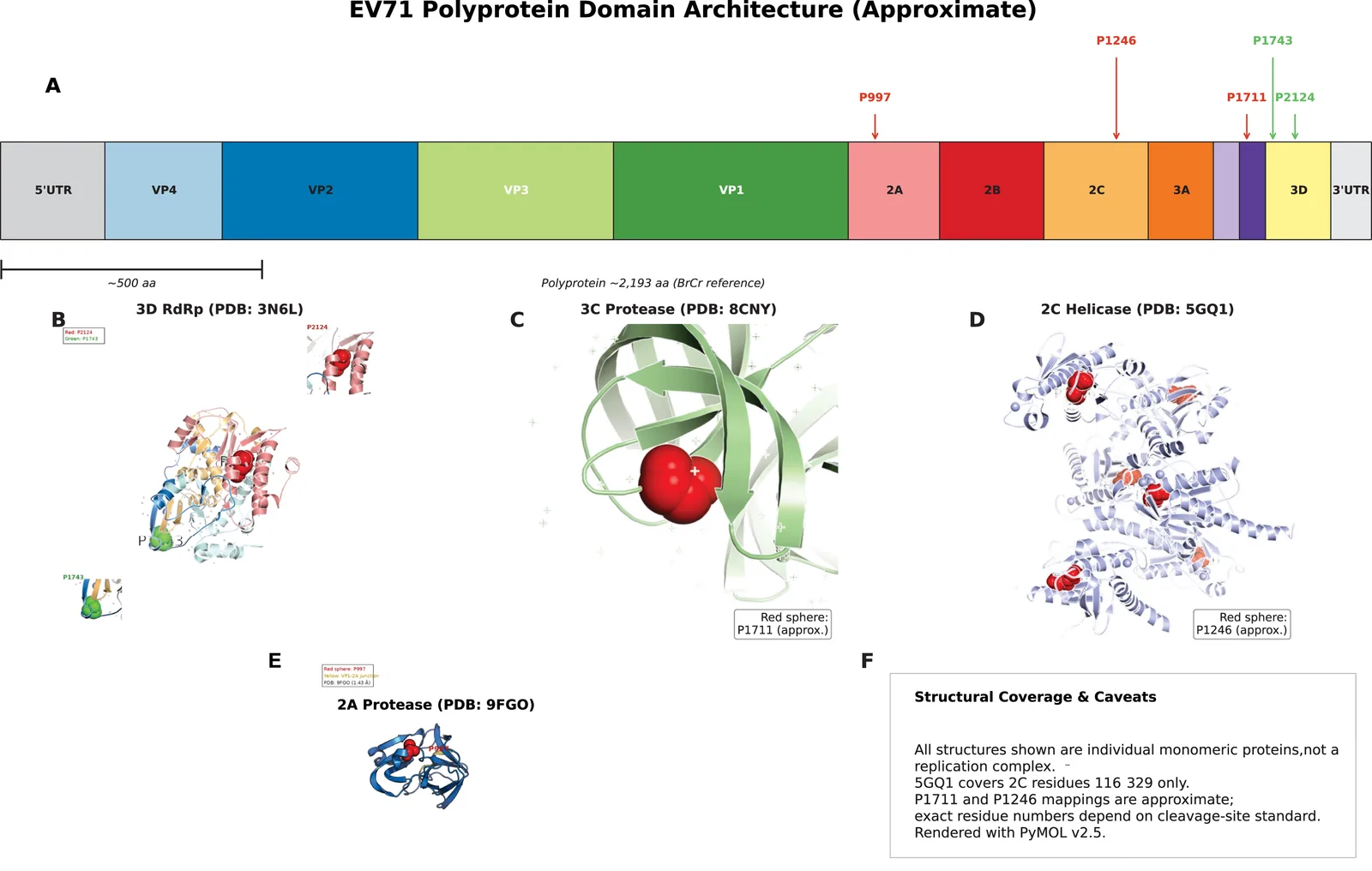
Structural localisation of the five neurovirulence-associated positions on individual experimentally determined EV-A71 protein structures. This is a composite illustration, not an experimentally determined multi-protein complex. Each position was mapped onto its corresponding monomeric structure because no experimentally resolved replication-machinery complex is currently available. The polyprotein schematic (panel A) is approximate and does not include the 3’UTR or the full C-terminal extension of 3D RdRp. Mapping of P1246 and P1711 to mature-protein residues is approximate; exact residue numbers depend on the cleavage-site standard used. P997 falls within 2 A and has no experimental structure. (A) 3D RdRp (PDB: 3N6L): P2124 maps to residue 393 in the thumb-domain surface loop adjacent to helix 20. (B) 3C protease (PDB: 8CNY): P1711 maps approximately to residue 163 in a C-terminal flexible loop. (C) 3D RdRp N-terminal vicinity: P1743 maps to residue 12 in the index-finger subdomain. (D) 2C helicase (PDB: 5GQ1, residues 116–329): P1246 maps approximately to residue 135 within the ATPase/helicase domain. (E) P997 falls within 2 A under standard BrCr cleavage-site annotation and was not assigned to an experimental structure. Insets show 2×magnified views.

Three-dimensional structural mapping of the five positions was paired with BrCr reference-strain mature-protein numbering so that each polyprotein coordinate can be interpreted at the protein and residue level (Fig. 10; Table 5).

The mature-protein mapping shows that the strongest signal, P2124, lies in the 3D RdRp thumb-domain surface loop, whereas P1743 lies near the 3D N-terminal index-finger region, P1711 maps to a C-terminal loop of 3C protease, and P1246 maps within the resolved 2C ATPase/helicase domain. P997 is retained as a statistically selected polyprotein coordinate but is annotated as unresolved because standard BrCr cleavage sites place it within 2 A, for which no experimental EV-A71 structure is available.

**Table 5:** Mapping of the five core neurovirulence-associated positions from polyprotein coordinates to mature protein residues (BrCr reference strain, GenBank: U22521).

Polyprotein position	Mature protein	Residue (mature)	Amino acid (BrCr ref.)	Domain/Region	PDB structure
P2124	3D RdRp	393	Glu	Thumb domain, surface-exposed loop adjacent to helix 20	3N6L
P1743	3D RdRp	12	Ala	N-terminal region, index-finger subdomain	3N6L
P1711	3C Protease	∼163^a^	–	C-terminal flexible loop	8CNY
P1246	2C Helicase	∼135^a^	–	ATPase/helicase domain (residues 116–329)	5GQ1
P997	2A (unresolved)^b^	–	–	–	–

Note: All mature-protein assignments are approximate and based on the BrCr reference strain (GenBank: U22521). P1246 and P1711 residue numbers depend on the cleavage-site standard; P997 has no experimental structure. No multi-protein replication complex structure is available.

a Approximate residue number; exact mapping depends on the polyprotein cleavage-site standard used.

b Standard BrCr cleavage sites place P997 within the 2A protein; the original assignment to 2C/3A requires independent validation. No experimental structure is available for EV-A71 2A.

## 4 Discussion

This study identifies a compact physicochemical signature associated with EV-A71 neurovirulence across a globally diverse collection of full-length genomes. Rather than treating amino-acid substitutions as isolated categorical events, we asked whether clinically severe isolates share recurrent changes in residue properties at homologous positions. The answer was unexpectedly parsimonious: from thousands of Z-scale descriptors, five polyprotein coordinates were sufficient to capture much of the separation between CNS-associated and non-CNS isolates. This finding does not imply that EV-A71 neurovirulence is reducible to five residues alone. Rather, it suggests that a small number of recurrent biophysical perturbations can act as observable markers of a broader evolutionary state associated with CNS-involved isolates, without by itself proving increased viral fitness in the CNS or in any other tissue.

The interpretation of this signature is governed by the central constraint of the study: only seven CNS-associated full-length genomes were available. This positive-class scarcity is not a secondary technical issue but the premise under which all statistical screening, LOOCV benchmarking, SHAP attribution, phylogenetic interpretation, and nomogram construction must be read. Consequently, the present results should be considered hypothesis-generating associations that prioritise candidate residues for follow-up, not validated determinants of EV-A71 neurovirulence.

The location of these positions is central to cautious interpretation. Previous work on EV-A71 virulence has naturally emphasised capsid determinants such as VP1-Q145E and VP1-E164D, as well as regulatory changes in the 5${}^{\prime }$UTR (Yeh et al., 2011, Zaini and McMinn, 2012, Chua et al., 2008). Our analysis instead places the strongest statistical signals in the P2–P3 region, including mature proteins involved in RNA synthesis, proteolytic processing, and replication-complex biology. BrCr-based mapping assigned P2124 to residue 393 of 3D RdRp, in a thumb-domain surface loop adjacent to helix 20, and assigned P1743 to the N-terminal index-finger region of the same polymerase. P1711 mapped to a C-terminal loop of 3C protease, P1246 to the resolved ATPase/helicase domain of 2C, and P997 to 2 A, for which no experimental EV-A71 structure is currently available. These structural locations provide biochemical context for follow-up experiments, but they do not demonstrate that the identified physicochemical shifts change viral replication, tissue tropism, or neurovirulence.

Among the five positions, P2124_Z1 had the largest odds ratio and SHAP attribution in the fitted model. Its mapping to a solvent-accessible region of 3D RdRp makes hydrophobicity a reasonable biochemical feature to examine, because substitutions at exposed polymerase surfaces could in principle affect local packing, solvent exposure, or protein–protein contacts (Wang et al., 2012, Tan et al., 2013, Ferrer-Orta et al., 2006). However, the present sequence-based analysis does not test polymerase activity, protein interaction strength, membrane association, or replication kinetics. The P1246_Z3 association should be interpreted even more cautiously. This position maps approximately to residue 135 of 2C, within the ATPase/helicase domain covered by PDB 5GQ1, and the statistical signal indicates only that lower polarity at this coordinate was associated with CNS-involved isolates in the present dataset. The result does not establish altered 2C function, replication-organelle biology, neural-cell tropism, or any CNS-selective replication mechanism. A conservative interpretation is that altered polarity at this 2C position may mark a local electrostatic or conformational hypothesis for future mutagenesis and biochemical assays; at present, it is not a confirmed mechanism.

The performance profile of the machine-learning models also informs the biology. Logistic regression outperformed more flexible ensemble and kernel methods under leave-one-out cross-validation, achieving an AUC of 0.889 despite the small number of CNS-confirmed genomes. In a dataset with only seven positive samples, this result should not be read as evidence that the underlying biology is simple. Nor should the LOOCV AUC be interpreted as a stable estimate of prospective accuracy, because the addition or removal of one CNS genome could materially change sensitivity, ROC geometry, and feature weights. Instead, it indicates that the measured signal is approximately monotonic in the five-dimensional Z-scale space: increasing hydrophobicity at P2124 and P997, increased volume at P1743, reduced hydrophobicity at P1711, and reduced polarity at P1246 shifted predictions in consistent directions. The success of a sparse linear model is therefore useful precisely because it resists overfitting and yields interpretable coefficients that can be audited biologically. In this setting, model transparency is not a secondary feature; it is what allows statistical discrimination to be translated into experimentally testable hypotheses rather than asserted mechanisms.

The phylogenetic analysis further argues against a lineage-specific explanation. CNS-associated isolates were scattered across divergent branches rather than forming a single monophyletic cluster, implying that similar physicochemical profiles can arise independently on different genetic backgrounds. This pattern is compatible with convergent evolution, but the inference is necessarily provisional because it rests on seven CNS-associated genomes. A practical implication is that surveillance based only on genotype labels or previously described single mutations may miss variants with similar physicochemical profiles. A physicochemical screen, by contrast, can recognise different amino-acid substitutions that produce similar hydrophobicity, volume, or polarity shifts at the same mapped positions. Such a framework is not intended to replace epidemiological, clinical, or experimental evidence, and it should be used only as an early prioritisation layer for newly sequenced isolates.

Several limitations temper these conclusions, and the most important is the very small positive class: only seven full-length CNS-associated genomes with suitable metadata were available in public databases, compared with 260 non-CNS genomes. This 7:260 imbalance increases the risk of statistical false positives, makes LOOCV AUC estimates potentially optimistic and unstable, and limits the generalisability of the classifier. Leave-one-out validation, class weighting, and convergent evidence from odds ratios, SHAP values, structural mapping, and phylogeny help organise the available evidence, but they cannot substitute for independent validation. The scarcity of CNS full-length genomes is biologically and practically understandable: EV-A71 CNS complications are rare relative to uncomplicated HFMD; routine clinical sequencing often targets VP1 rather than the whole genome; CSF viral abundance can be extremely low, as illustrated by the 0.06–0.55 reads-per-million EV-A71 range reported by Leon et al. (2020); and many GenBank entries lack explicit neurological phenotype annotations. Therefore, although the seven CNS genomes constitute one of the larger publicly retrievable phenotype-anchored EV-A71 CNS full-genome sets available for this type of analysis, they remain the greatest constraint on inference. The current model, five-position signature, structural interpretation, and nomogram should be viewed as hypothesis-generating. Prospective validation in larger, clinically annotated, and more balanced independent cohorts is urgently needed before these markers can be used for robust surveillance, risk prediction, or mechanistic claims. The binary CNS/non-CNS phenotype also compresses a spectrum of neurological disease into a single label, potentially obscuring determinants that distinguish meningitis, encephalitis, acute flaccid paralysis, and fatal cardiopulmonary involvement. In addition, the coordinate system used here is alignment based and offset from canonical BrCr numbering. Mature-protein assignments, especially P1246 to 2C and P997 to 2 A, should therefore be regarded as approximate where cleavage-site boundaries are uncertain. Finally, Z-scales capture three major physicochemical axes but not all relevant features of protein evolution, such as backbone flexibility, local epistasis, RNA secondary structure, or host-specific immune selection.

Taken together, these results support a cautious view that EV-A71 CNS involvement may be statistically associated with physicochemical shifts in non-structural protein regions, rather than demonstrating replication-machinery adaptation or causality. Within the limits of seven CNS-positive genomes, the five-position signature provides an interpretable bridge between viral genome surveillance and structural hypothesis generation. Its current value lies in hypothesis generation and risk prioritisation; its longer-term value will depend on prospective validation in larger and more balanced clinical cohorts and direct experimental testing of the mapped residues in infectious clones or biochemical systems. If confirmed, this approach could complement conventional genotype-based surveillance by identifying variants with convergent physicochemical profiles even when they are phylogenetically distant.

## 5 Conclusions

This study suggests that EV-A71 CNS involvement may be associated not merely with discrete amino acid identities but with continuous physicochemical properties of residues at five positions within the non-structural polyprotein. A parsimonious logistic regression model built on Z-scale descriptors (hydrophobicity, volume, polarity) achieved an internal LOOCV AUC of 0.889; however, this estimate is constrained by the availability of only seven CNS-positive genomes and should not be interpreted as definitive evidence of generalisable prediction. SHAP attribution identified P2124_Z1 (hydrophobicity at 3D RdRp residue 393) as the dominant model contributor and P1246_Z3 (polarity at approximately 2C helicase residue 135) as the feature most strongly associated with lower CNS odds, but these are statistical and model-attribution findings rather than confirmed mechanisms. BrCr-based mature-protein mapping places the selected positions in structurally interpretable contexts and generates testable biochemical hypotheses; it does not establish altered replication-organelle formation, neural tropism, or CNS-selective replication. Phylogenetic dispersion of the seven CNS-associated strains across divergent lineages is compatible with convergent evolution, but this interpretation requires confirmation as additional CNS full-length genomes become available. The nomogram and risk-scoring framework should therefore be used only for exploratory prioritisation at this stage, while the identified positions require prospective cohort validation and direct experimental testing.

## CRediT authorship contribution statement

**Xiaoqian Wang:** Writing – original draft, Visualization, Validation, Software, Methodology, Investigation, Formal analysis, Data curation. **Feng Li:** Writing – review & editing, Funding acquisition.

## Informed consent

Not applicable. This study analysed publicly available genomic data without involvement of human participants.

## Declaration of competing interest

The authors declare that they have no known competing financial interests or personal relationships that could have appeared to influence the work reported in this paper.

## Data Availability

All sequence data analysed in this study are publicly available through GenBank ( https://www.ncbi.nlm.nih.gov/genbank/). The curated dataset with clinical annotations, analysis scripts, and trained model parameter files are publicly available at GitHub: https://github.com/biologist-wang/EV71-Prediction.

## Appendix A Supplementary data

The following supporting information can be downloaded at: Supplementary material 1 provides the complete list of 20 physicochemical positions reaching significance (P<0.001) in Mann–Whitney U screening; Supplementary material 2 are the hyperparameter settings for all eight machine learning algorithms. GenBank accession numbers for all 267 sequences are listed in Supplementary material 3. Full data for the remaining 20 significant positions are listed in Supplementary material 4. The corresponding heatmap for the complete cohort of 267 samples is shown in Supplementary material 5. Supplementary material 6 provides a complete reproducibility parameter document for the machine-learning pipeline, including Z-scale descriptors for the 20 standard amino acids (Table S1), feature engineering and encoding parameters (Table S2), two-stage feature-selection parameters including statistical thresholds, collinearity filtering, and domain-context filtering criteria (Table S3), machine-learning model hyperparameters (Table S4), cross-validation and evaluation protocol (Table S5), and software environment/version information (Table S6). All parameters in Supplementary material 6 were extracted directly from the analysis scripts (Aio.py and rebuild_analysis_v3.py) to facilitate full reproducibility. Supplementary material 7 provides enlarged views of Fig. 10 subpanels B–E to improve visualisation of the mapped sites: P1711 in 3C protease, P1743 in the 3D RdRp N-terminal vicinity, P1246 in 2C helicase, and the schematic 2 A location of P997.

MMC S1 Complete list of 20 physicochemical positions significant in Mann–Whitney U screening (P < 0.001). MMC S1

MMC S2 Hyperparameter settings for the eight machine-learning algorithms. MMC S2

MMC S3 GenBank accession numbers for all 267 EV71 sequences included in the study. MMC S3

MMC S4 Full data for the 20 significant physicochemical positions identified in the screening. MMC S4

MMC S5 Heatmap of the complete cohort of 267 EV71 samples across the 20 significant positions. MMC S5

MMC S6 Reproducibility parameters for feature engineering, model training, validation, and software environment. MMC S6

MMC S7 Enlarged views of structural mapping panels for P1711, P1743, P1246, and P997. MMC S7
