# Supplementary material for: Physicochemical fingerprinting reveals convergent evolutionary determinants of enterovirus A71 neurovirulence through integrative machine learning and structural analysis
Source: Virus Res. 2026 Jul 22;371:199772. doi: 10.1016/j.virusres.2026.199772 (PMC13522892; doi:10.1016/j.virusres.2026.199772)
Supplement: Supplementary file 1 [file mmc5.pdf]

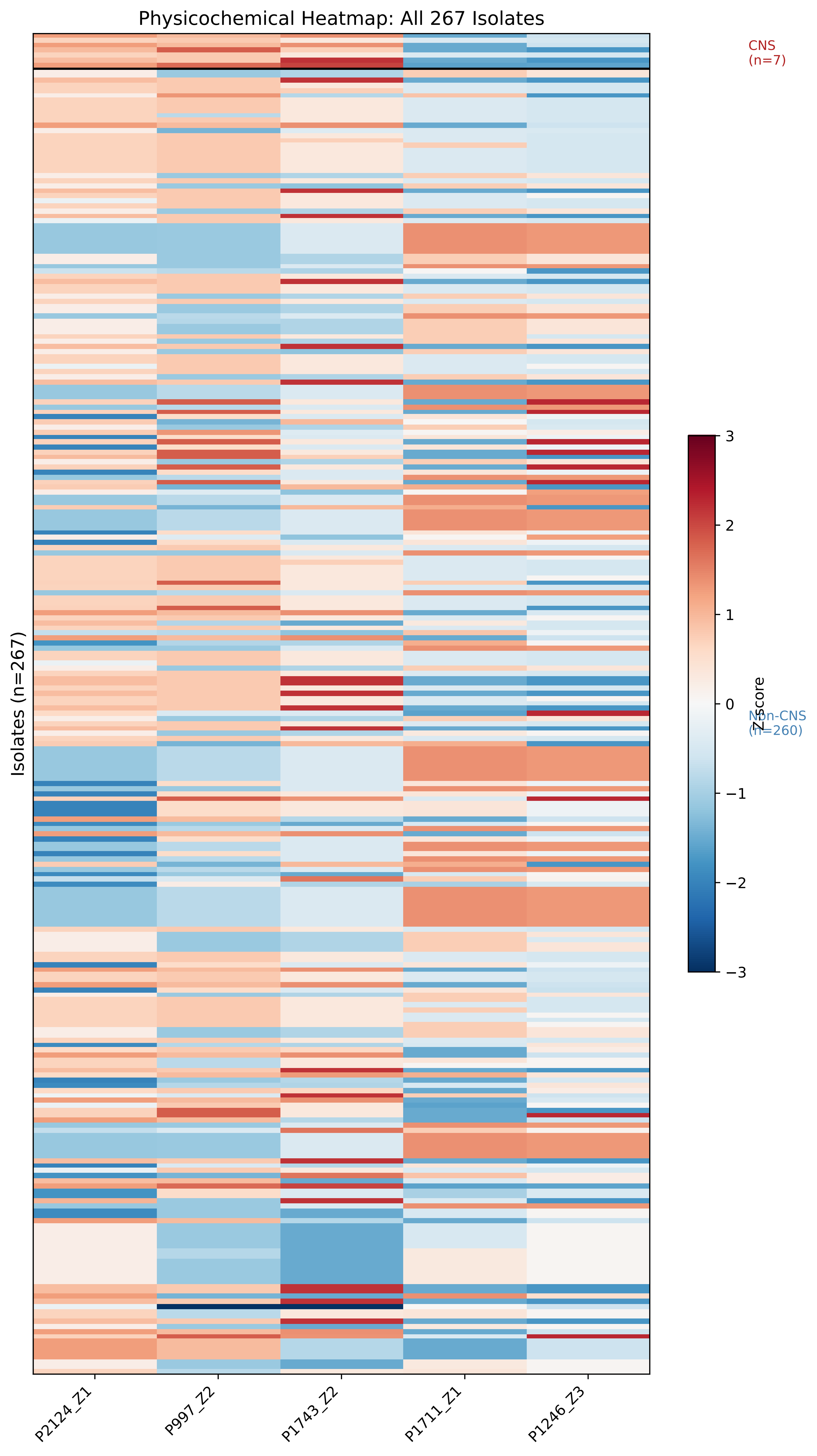

Physicochemical Heatmap: All 267 Isolates
CNS
(n=7)
Non-CNS
(n=260)
Isolates (n=267)
P2124_Z1
P997_Z2
P1743_Z2
P1711_Z1
P1246_Z3
Z score
3
2
1
0
−1
−2
−3
