# Supplementary material for: Physicochemical fingerprinting reveals convergent evolutionary determinants of enterovirus A71 neurovirulence through integrative machine learning and structural analysis
Source: Virus Res. 2026 Jul 22;371:199772. doi: 10.1016/j.virusres.2026.199772 (PMC13522892; doi:10.1016/j.virusres.2026.199772)
Supplement: Supplementary file 2 [file mmc4.docx]

*Association statistics for the 15 additional significant positions (ranked 6–20 by P value) not shown in Figure 4*

*Note. The top five positions (rank 1–5) are presented in Figure 4.. Residue numbers marked with "~" are approximate and depend on the polyprotein cleavage-site standard used. Structural protein positions (VP4–VP1) are not assigned individual residue numbers owing to variable cleavage sites across enterovirus serotypes. Z-scale properties: Z1 = hydrophobicity, Z2 = bulkiness, Z3 = polarity. OR = odds ratio; CI = confidence interval; IQR = interquartile range.*

| **Rank** | **Polyprotein position** | **Z-scale** | **Property** | **Mature protein** | **Residue (mature)** | **CNS group (median, IQR)** | **Non-CNS group (median, IQR)** | **P value** | **OR (95% CI)** | **Note** |
| --- | --- | --- | --- | --- | --- | --- | --- | --- | --- | --- |
| 6 | P2061 | Z1 | Hydrophobicity | 3D RdRp | 331 | 2.23 (2.07-3.03) | 0.07 (-1.22-2.18) | 0.00208 | 3.15 (1.24-8.00) | nan |
| 7 | P1411 | Z1 | Hydrophobicity | 2C Helicase | ~301 | -2.69 (-4.19--0.57) | 0.71 (0.07-1.96) | 0.0021 | 0.59 (0.41-0.83) | approximate |
| 8 | P1319 | Z2 | Bulkiness | 2C Helicase | ~209 | 1.13 (0.05-2.42) | -1.03 (-1.68--1.03) | 0.00216 | 1.99 (1.26-3.13) | approximate |
| 9 | P1093 | Z1 | Hydrophobicity | 2B | ~87 | 2.23 (2.21-2.55) | 0.07 (0.07-2.18) | 0.00228 | 2.97 (1.15-7.66) | approximate |
| 10 | P611 | Z2 | Bulkiness | Structural protein (VP4–VP1) | — | 1.45 (0.46-1.98) | 0.39 (-2.53-0.39) | 0.00237 | 2.37 (1.20-4.70) | nan |
| 11 | P282 | Z3 | Polarity | Structural protein (VP4–VP1) | — | 0.57 (-0.34-1.60) | -1.03 (-3.14--0.98) | 0.00243 | 1.94 (1.21-3.12) | nan |
| 12 | P377 | Z1 | Hydrophobicity | Structural protein (VP4–VP1) | — | 2.88 (2.51-3.15) | 0.92 (-2.69-2.18) | 0.00262 | 4.42 (1.06-18.44) | nan |
| 13 | P2298 | Z1 | Hydrophobicity | 3D RdRp | — | 3.08 (1.11-3.64) | 0.00 (-4.19-0.92) | 0.0027 | 1.72 (1.12-2.64) | Within 3D RdRp region (P-number coordinate) |
| 14 | P354 | Z1 | Hydrophobicity | Structural protein (VP4–VP1) | — | -2.69 (-3.72--0.57) | 0.07 (0.07-2.23) | 0.00281 | 0.64 (0.47-0.86) | nan |
| 15 | P398 | Z1 | Hydrophobicity | Structural protein (VP4–VP1) | — | -1.39 (-4.68--1.22) | 1.96 (-1.22-2.84) | 0.00302 | 0.67 (0.49-0.91) | nan |
| 16 | P1406 | Z1 | Hydrophobicity | 2C Helicase | ~296 | 2.84 (2.18-3.22) | 0.00 (-4.19-2.18) | 0.00324 | 2.14 (1.06-4.33) | approximate |
| 17 | P565 | Z3 | Polarity | Structural protein (VP4–VP1) | — | -1.40 (-2.42--1.14) | 0.09 (-0.98-0.30) | 0.00328 | 0.51 (0.30-0.86) | nan |
| 18 | P2337 | Z2 | Bulkiness | 3D RdRp | — | 1.13 (0.56-1.35) | 0.00 (-1.73-0.53) | 0.0038 | 2.51 (1.30-4.85) | Within 3D RdRp region (P-number coordinate) |
| 19 | P1626 | Z3 | Polarity | 3C Protease | ~79 | -1.40 (-3.14--1.27) | 0.00 (-1.40-0.30) | 0.00399 | 0.38 (0.21-0.72) | approximate |
| 20 | P394 | Z1 | Hydrophobicity | Structural protein (VP4–VP1) | — | -2.69 (-4.75--2.59) | 2.23 (-2.69-2.23) | 0.00407 | 0.54 (0.33-0.88) | nan |
