# Supplementary material for: Physicochemical fingerprinting reveals convergent evolutionary determinants of enterovirus A71 neurovirulence through integrative machine learning and structural analysis
Source: Virus Res. 2026 Jul 22;371:199772. doi: 10.1016/j.virusres.2026.199772 (PMC13522892; doi:10.1016/j.virusres.2026.199772)
Supplement: Supplementary file 3 [file mmc6.docx]

**Supplementary Tables S1–S6: Detailed Method Parameters**

**This document provides the complete set of parameters used in the machine-learning pipeline for EV71 CNS-invasion risk prediction.** All parameters are extracted directly from the analysis code (Aio.py, rebuild_analysis_v3.py) to ensure full reproducibility.

# Table S1. Z-scale descriptors for the 20 standard amino acids

*Reference: Sandberg et al. (1998) J. Med. Chem. 41(14): 2481–2491. Each amino acid is encoded as a 3-dimensional vector [Z1, Z2, Z3].*

| Amino Acid | Z1 (Lipophilicity) | Z2 (Steric Bulk) | Z3 (Polarity) | 3-Letter Code |
| --- | --- | --- | --- | --- |
| A | 0.07 | -1.73 | 0.09 | Ala |
| R | 2.88 | 2.52 | -3.44 | Arg |
| N | 3.22 | 1.45 | 0.84 | Asn |
| D | 3.64 | 1.13 | 2.36 | Asp |
| C | 0.71 | -0.97 | 4.13 | Cys |
| E | 3.08 | 0.39 | -0.07 | Glu |
| Q | 2.18 | 0.53 | -1.14 | Gln |
| G | 2.23 | -5.36 | 0.30 | Gly |
| H | 2.41 | 1.74 | 1.11 | His |
| I | -4.44 | -1.68 | -1.03 | Ile |
| L | -4.19 | -1.03 | -0.98 | Leu |
| K | 2.84 | 1.41 | -3.14 | Lys |
| M | -2.49 | -0.27 | -0.41 | Met |
| F | -4.92 | 1.30 | 0.45 | Phe |
| P | -1.22 | 0.88 | 2.23 | Pro |
| S | 1.96 | -1.63 | 0.57 | Ser |
| T | 0.92 | -2.09 | -1.40 | Thr |
| W | -4.75 | 3.65 | 0.85 | Trp |
| Y | -1.39 | 2.32 | 0.01 | Tyr |
| V | -2.69 | -2.53 | -1.29 | Val |

# Table S2. Feature engineering and encoding parameters

| Encoding method | Z-scale (3D per residue) × polyprotein length |
| --- | --- |
| Polyprotein length | 2,193 amino acids (BrCr reference strain) |
| Total raw features | 2,193 × 3 = 6,579 Z-scale features |
| Missing residue handling | Z_SCALE.get(seq[i], [0, 0, 0]) — defaults to [0,0,0] if amino acid not found |
| Feature naming convention | P{position}_Z{dimension}, e.g., P997_Z2 |
| Data standardization | StandardScaler (z-score: mean=0, std=1) applied before model fitting |

# Table S3. Two-stage feature selection parameters

| Stage 1: Univariate test | Mann–Whitney U test (scipy.stats.mannwhitneyu) |
| --- | --- |
| Stage 1 alternative hypothesis | two-sided |
| Stage 1 raw P-value threshold | P < 0.05 |
| Multiple-testing correction | Bonferroni correction: p_adj = p_raw × n_features (n = 6,579) |
| Corrected threshold | p_adj < 0.05 |
| Stage 2: Collinearity filter | Spearman rank correlation \|ρ\| < 0.7 |
| Stage 2: Domain-context filter | Manual curation retaining functionally annotated positions |
| Final feature count (main model) | 5 positions × 3 dimensions = 15 features |
| Final feature list | P2124_Z1, P1246_Z3, P997_Z2, P1711_Z1, P1743_Z2 |

# Table S4. Machine learning model hyperparameters

All models implemented in Python 3 with scikit-learn (v1.3+) and XGBoost (v2.0+).

| Model | Key Hyperparameters | Class Imbalance Handling | sklearn Module |
| --- | --- | --- | --- |
| Logistic Regression | C=1.0, solver=liblinear, max_iter=100, penalty=l2, tol=1e-4 | class_weight=balanced | sklearn.linear_model.LogisticRegression |
| Random Forest | n_estimators=100, max_depth=None, min_samples_split=2, min_samples_leaf=1, criterion=gini | class_weight=balanced | sklearn.ensemble.RandomForestClassifier |
| SVM (RBF) | C=1.0, kernel=rbf, gamma=scale, probability=True, tol=1e-3 | class_weight=balanced | sklearn.svm.SVC |
| Extra Trees | n_estimators=100, max_depth=None, min_samples_split=2, criterion=gini | class_weight=balanced | sklearn.ensemble.ExtraTreesClassifier |
| Gradient Boosting | n_estimators=100, learning_rate=0.1, max_depth=3, loss=log_loss, subsample=1.0 | None (sample-weighting not used) | sklearn.ensemble.GradientBoostingClassifier |
| AdaBoost | n_estimators=100, learning_rate=1.0, algorithm=SAMME.R | None | sklearn.ensemble.AdaBoostClassifier |
| Ridge Classifier | alpha=1.0, solver=auto, tol=1e-4 | class_weight=balanced | sklearn.linear_model.RidgeClassifier |
| Gaussian Naïve Bayes | var_smoothing=1e-9 (default) | None (inherently handles via priors) | sklearn.naive_bayes.GaussianNB |

# Table S5. Cross-validation and evaluation protocol

| CV strategy | Leave-One-Out Cross-Validation (LOOCV) |
| --- | --- |
| CV rationale | Small sample size (n=267) with extreme class imbalance (CNS=7, Non-CNS=260) |
| Train/test split per fold | 266 training / 1 test |
| Total folds | 267 |
| Primary metric | AUC-ROC (Area Under the Receiver Operating Characteristic Curve) |
| Secondary metrics | Accuracy, Sensitivity (Recall), Specificity, F1-score, MCC |
| Binary threshold | 0.5 (default) for all models |
| Probability calibration | SVC: probability=True (Platt scaling); others: native predict_proba() |
| Random state | 42 (where applicable) |
| Negative class encoding | Label = 0 (Non-CNS) |
| Positive class encoding | Label = 1 (CNS) |

# Table S6. Software environment and versions

| Software / Package | Version | Purpose |
| --- | --- | --- |
| Python | 3.9+ | Programming language |
| NumPy | 1.24+ | Numerical computing |
| pandas | 2.0+ | Data manipulation |
| scikit-learn | 1.3+ | Machine learning models and metrics |
| SciPy | 1.11+ | Statistical tests (Mann–Whitney U) |
| XGBoost | 2.0+ | Gradient boosting (if used) |
| PyMOL | 3.2.0a | Molecular structure rendering |

# Notes on Reproducibility

**1. Default parameters:** Unless explicitly listed in Table S4, all model parameters use the scikit-learn default values.

**2. Feature scaling:** StandardScaler is applied per-fold during LOOCV to prevent data leakage. Tree-based models (Random Forest, Extra Trees, Gradient Boosting, AdaBoost) and Naïve Bayes are trained on unscaled features.

**3. Bonferroni correction:** The corrected P-value is computed as p_adj = p_raw × 6,579. Features with p_adj < 0.05 are retained for Stage 2 filtering.

**4. Class imbalance:** The CNS class comprises only 7 out of 267 samples (2.6%). class_weight=balanced is used for parametric models to mitigate bias toward the majority class.

**5. SHAP analysis:** SHAP values are computed post-hoc on the full dataset using the best-performing model to identify feature importance rankings.
