# Supplementary material for: Physicochemical fingerprinting reveals convergent evolutionary determinants of enterovirus A71 neurovirulence through integrative machine learning and structural analysis
Source: Virus Res. 2026 Jul 22;371:199772. doi: 10.1016/j.virusres.2026.199772 (PMC13522892; doi:10.1016/j.virusres.2026.199772)
Supplement: Supplementary file 4 [file mmc7.docx]

**Supplementary Subfigure of Figure 10**

**This section provides enlarged views of Figure 10 subpanels B--E to improve visualisation of the mapped sites: P1711 in 3C protease, P1743 in the 3D RdRp N-terminal vicinity, P1246 in 2C helicase, and the schematic 2A location of P997.**
